# The Question of the Registration Fee

**Published:** 1909-04-03

**Authors:** 


### NURSING ADMINISTRATION.

### THE QUESTION OF THE REGISTRATION FEE.

Few matters have aroused more attention in the controversies centring round the registration of nurses than the amount of the registration fee. Nobody can be quite sure how many nurses would offer themselves for registration, and it is, therefore, next door to impossible to form a correct estimate of the fees which would be paid. But the cost of registration is not so difficult to estimate. It must, however, be remembered that registration and examination are two very different processes, and that the amount charged for one would certainly not suffice for both. In Lord Ampthill's Bill provision has been made for both examination and registration, and a maximum fee of five guineas has been provided for. This does not, of course, mean that the Central Board would be compelled to charge this amount. It merely means that it could not charge more. Experience shows, however, that the fees which are permissible will be the fees imposed. The Scotch Registration Bill, on the other hand, imposes a maximum of two guineas for registration, but this is on the basis of the examinations being conducted as now in the various training schools and not by a central authority. In the one case the nurses pay the cost of their examination; in the other case the cost is borne by the training school. The matter is one of considerable importance to nurses. Ever since the first establishment of training schools for nurses the privilege of being examined and certificated at the expense of the institution has been enjoyed by nurses. We have often pointed out that the cost of lecturers and examiners is no inconsiderable item in the expenses of probationers. What it amounts to in the aggregate is now ascertained, seeing that the cost of the final or qualifying examination alone is estimated to work out at three guineas. In first rate training schools the process of examination is going on all through the probationer's course, and it may be reckoned that it costs the institution from first to last at least five guineas a head. If the onus of bearing the cost of examinations is thrown on the nurses it will impose a yearly collective charge of at least £6,000 on probationers completing their course. For the number of probationers turned out every year from hospitals containing over 100 beds is approximately 2,000, without counting Poor-law infirmaries. It is interesting to consider the expenses of both registration and examinations.

1. Registration.—The expenses of the department would certainly not exceed the following estimates:

Publication of Register, postage, printing, etc. (cost of corrections being defrayed by nurses).

Rent of offices, salaries of registrar, clerks, etc.

Travelling expenses, etc., of Board, consisting of 15 persons, sitting twice a week for 40 weeks.

A steady rate of 2,000 nurses registering at two guineas apiece would certainly defray these expenses and leave a good margin.

2. Examination.—The expenses would fall under the following headings:

Hire of rooms, cost of postage, stationery, printing, etc.
Cost of setting papers.
Cost of conducting examinations.
Cost of correcting papers.
Cost of conducting *viva voce*.

When it is remembered that the candidates would have at least six subjects to pass in, in addition to *viva voce* and practical demonstrations of efficiency, it is clear that the expense would be considerable. The Scotch authorities seem to be contemplating that the institution would continue to bear the cost of examining their own nurses, and it is probable that they would continue to do so even under the new conditions imposed by the Nursing Council. As in lieu of examiners appointed from among members of the staff, and undertaking their duties for merely nominal remuneration as part of their ordinary work, there would under the registration scheme be outside paid examiners appointed by the Council "to regulate and supervise" the examinations they could not fail to be far more costly than under the present independent plan, although in many respects they would be less costly than if they were altogether dissociated from the training schools.

It is hardly necessary to urge, at the present moment, the advantage to the training school of employing outside examiners. Apart from the objections which can be urged against the practice of "branding your own herrings," there are objections from the nurses' point of view. It is not every hospital which contains men on its staff trained for examination work. It needs a combination of qualities and talents to make good examiners, and a large amount of practice is essential in testing women unaccustomed to express themselves concisely either verbally or on paper. Moreover, the outside examiner can be of the greatest possible value to the head of the training school by indicating strong or weak points in the report, and in making suggestions as to the preparation of candidates. It would be indispensable that the examiners in the nursing subjects should be women, not necessarily medical practitioners, but such as have had experience in training nurses themselves under the best conditions, and with wide knowledge of varied training schools. The right kind of examiners both male and female could prove of the utmost support and assistance to the matrons whose candidates they examined, acting in this way as the examiners do at the public schools for the Oxford and Cambridge Board Examinations. The value of the examination would thus be felt far beyond the sphere of the individual candidates.

It should be clearly understood that the cost of holding the examinations in the training schools instead of at one or two separate centres would not merely lead to a saving of expense, but would also —and this is the important point for nurses—cause the expense to fall mainly on the institution instead of on the candidates.

